# Multivessel versus Single Vessel Angioplasty in Non-ST Elevation Acute Coronary Syndromes: A Systematic Review and Metaanalysis

**DOI:** 10.1371/journal.pone.0148756

**Published:** 2016-02-17

**Authors:** Javier Mariani, Alejandro Macchia, Maximiliano De Abreu, Gabriel Gonzalez Villa Monte, Carlos Tajer

**Affiliations:** 1 Cardiology Department, Hospital El Cruce “Néstor Carlos Kirchner”, Av. Calchaquí 5401 (B1888AAE), Florencio Varela, Buenos Aires, Argentina; 2 Fundación GESICA, Av. Rivadavia 2358 (C1034ACP), Ciudad Autónoma de Buenos Aires, Argentina; University Hospital Medical Centre, GERMANY

## Abstract

**Background:**

Multivessel disease is common in acute coronary syndrome patients. However, if multivessel percutaneous coronary intervention is superior to culprit-vessel angioplasty has not been systematically addressed.

**Methods:**

A metaanalysis was conducted including studies that compared multivessel angioplasty with culprit-vessel angioplasty among non-ST elevation ACS patients. Since all studies were observational adjusted estimates of effects were used. Pooled estimates of effects were computed using the generic inverse of variance with a random effects model.

**Results:**

Twelve studies were included (n = 117,685). Median age was 64.1 years, most patients were male, 29.3% were diabetic and 36,9% had previous myocardial infarction. Median follow-up was 12 months. There were no significant differences in mortality risk (HR 0.79; 95% CI 0.58 to 1.09; I^2^ 67.9%), with moderate inconsistency. Also, there were no significant differences in the risk of death or MI (HR 0.90; 95% CI 0.69 to 1.17; I^2^ 62.3%), revascularization (HR 0.76; 95% CI 0.55 to 1.05; I^2^ 49.9%) or in the combined incidence of death, myocardial infarction or revascularization (HR 0.83; 95% CI 0.66 to 1.03; I^2^ 70.8%). All analyses exhibited a moderate degree of inconsistency. Subgroup analyses by design reduced the inconsistency of the analyses on death or myocardial infarction, revascularization and death, myocardial infarction or revascularization. There was evidence of publication bias (Egger’s test p = 0.097).

**Conclusion:**

Routine multivessel angioplasty in non-ST elevation acute coronary syndrome patients with multivessel disease was not superior to culprit-vessel angioplasty. Randomized controlled trials comparing safety and effectiveness of both strategies in this setting are needed.

## Introduction

Current clinical practice guidelines recommend an invasive approach for patients with intermediate and high-risk features presenting with non-ST elevation acute coronary syndromes (NSTE-ACS) [1,2]. Since approximately 40–60% of NSTE-ACS patients who undergo coronary angiography, have multivessel coronary artery disease, treating physicians often face the decision of choosing the best revascularization strategy [3,4]. In cases where anatomy is suitable for percutaneous coronary intervention (PCI), and there is no clear indication of surgical revascularization, the decision usually stands between multivessel PCI (MV PCI) and culprit-vessel PCI (CV PCI). In such situations, AHA/ACC guidelines recommend that “a strategy of multivessel PCI, in contrast to culprit lesion–only PCI, may be reasonable in patients undergoing coronary revascularization as part of treatment for NSTE-ACS” [2].

Complete revascularization has the potential to improve outcomes by reducing recurrent events, particularly urgent revascularization procedures [5]. Nevertheless, these benefits could be offset by an increase in the risk of periprocedural myocardial infarction (MI), stent thrombosis, bleeding and contrast-induced nephropathy associated with MV PCI [6–8]. Furthermore, it has been suggested that MV PCI has lower procedure success rates than CV PCI [9].

In this study, the aim was to assess the evidence that compares MV PCI versus CV PCI among patients with NSTE-ACS with multivessel coronary artery disease through a systematic review and meta-analysis.

## Materials and Methods

The study protocol is registered in the international prospective register of systematic reviews (PROSPERO), number CRD42014015531 (available at http://www.crd.york.ac.uk/PROSPERO/display_record.asp?ID=CRD42014015531).

### Eligibility criteria

Studies were eligible if compared a revascularization strategy based on CV PCI (or one vessel only) versus MV PCI, among NSTE-ACS (MI or unstable angina) patients with multivessel coronary artery disease and with available outcome data for the analyses.

Only published articles were considered and there were no restrictions regarding design or language. Studies were included only if separated data was available for patients matching our target population.

Studies reporting data on patients with ST elevation MI and/or patients with cardiogenic shock, and those that compared PCI versus coronary artery bypass grafting (CABG) were excluded.

### Search strategy

We searched in MEDLINE (via PubMed, with no date restrictions), EMBASE (from 1980 to present) and PsycINFO (from 1987 to present). The terms used for electronic search were: [coronary angioplasty OR percutaneous coronary intervention OR pci OR revascularization] AND [(unstable angina) OR (myocardial infarction AND non st elevation) OR (non st elevation AND acute coronary syndrome)] AND [multivessel].

As recommended, reference lists of relevant studies and other published reviews on this issue were handsearched for potential studies [10].

### Data extraction

Two of the authors (J.M. and A.M.) assessed independently the articles retrieved by the search in an unblinded fashion. Eligibility was initially evaluated through revision of titles and abstracts and, when inclusion criteria were met or there were no clear exclusion criteria present, full texts were retrieved for further evaluation.

Since all studies were observational, to record data MOOSE guidelines were followed [10]. The extracted data from each study report included authors, year of publication, design, statistical methods for confounding control, loss in follow-up, follow-up duration, total number of patients registered, number of patients finally included in the analyses, cardiovascular risk factors, angiographic data, use of drug eluting stents (DES), outcome event data and adjusted estimates of effects. Data were collected in an *ad-hoc* case report form and then entered in a dedicated database.

All discrepancies were solved by consensus with the participation of a third author (C.T.).

For quality evaluation, it was computed the Newcastle-Ottawa Scale (NOS) as the sum of stars of each study [11]. The scale assigned a maximum of nine points, with more points indicating better quality. As recommended elsewhere, assessment of quality included the NOS but was not limited to it [10,12].

### Outcomes

The outcomes of interest were all cause mortality, death or MI, revascularization and the combined incidence of death, MI or revascularization. In all cases, definitions of events were maintained as reported in the original articles with no attempt to re-classify events.

### Statistics

The main analyses were conducted using the adjusted estimators of effects for each study (i.e. measures of effect obtained after controlling for confounders), and these were pooled with a random effects model using the generic inverse variance method, as described by DerSimonian and Laird [10,13]. To evaluate the influence of confounders on estimates of effects, we also conducted exploratory analyses using the raw data (i.e. number with events and number of patients in each study group). Individual and pooled adjusted estimates of effect were reported as hazard ratios (HR) with the corresponding 95% confidence intervals (95% CI); there were, however, four studies that reported the adjusted estimates as odds ratios (OR). In these cases, the ORs were converted to relative risks, as suggested elsewhere, and pooled in this way in a sensitivity analysis [9,14–17].

Heterogeneity was evaluated through the I^2^ statistic, which represents the percentage of variation between estimates of effects that cannot be explained by the play of chance; a value >50% was considered an indicator of moderate inconsistency and a value >75% as substantial inconsistency [18]. Possible sources of heterogeneity were explored in subgroup analyses that were defined by study designs, duration of follow-up, percentage of DES utilization and quality of the study determined by the NOS.

Publication bias was evaluated by the visual exploration of funnel plot, and formally through the Egger’s test, with p<0.1 considered as an indicator of a statistically significant asymmetry of the funnel plot [19].

All analyses were conducted using the R software and the ***meta*** package (the R Foundation for Statistical Computing, Vienna, Austria) [20].

## Results

### Included studies

**Fig 1:**
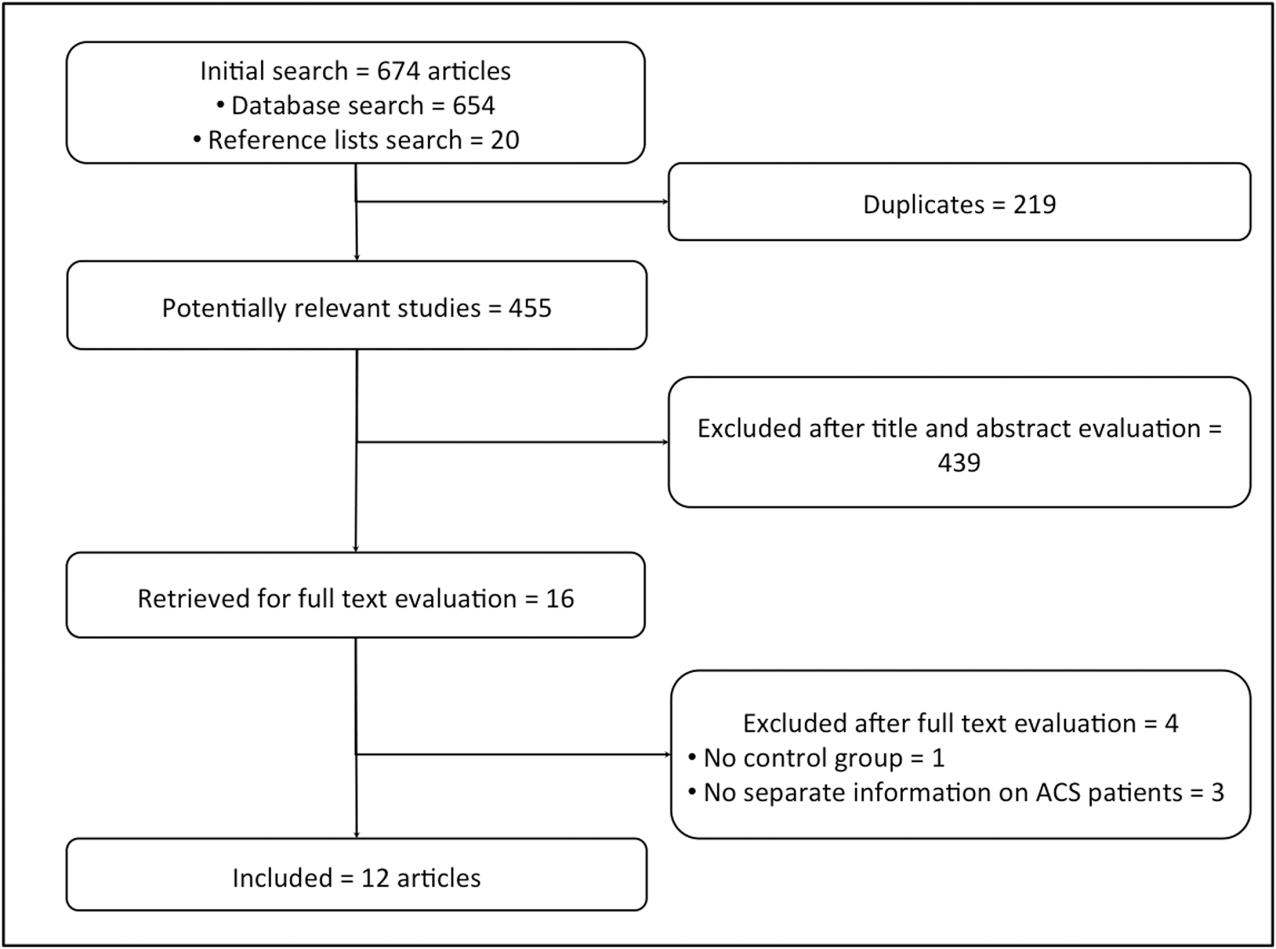
Flow chat of studies.

The initial search identified 674 articles, 219 of which were duplicates. After revision of the remaining titles and abstracts, 16 full texts were retrieved for further evaluation (Fig 1). One was excluded because there was no control group for comparison and three reported ACS patients jointly with stable chronic angina patients [21–24]. In all, 12 studies were included with reported data of 117,685 patients (38,477 received MV PCI and 79,208 received CV PCI) (Table 1).

**Table 1:** Characteristics of included studies.

Authors	Acromyn	Year	Countries	Design	Statistical adjustment	Inclusion criteria	Intervention definition	Control definition	Exclusion criteria	N in database	N analysed	N lost in follow-up
Bauer et al [14]	EHS-PCI	2011	Europe	Observational registry	Multivariate analyses (logistic regression)	Haemodynamically stable ACS and at least two epicardial vessel with ≥70% obstruction	PCI in ≥2 vessels	PCI in 1 vessel	Prior CABG. LM lesion	47407	1920	NA
Onuma et al [30]	RESEARCH—T-SEARCH	2013	Netherlands	Observational registry	Multivariate analyses (Cox)	NSTE-ACS and multivessel disease	PCI in ≥2 vessels	PCI in 1 vessel	Prior CABG. staged PCI	1312	990	40
Lee et al [29]		2011	Korea	Observational registry	Multivariate analyses (Cox)	NSTE-ACS. multivessel disease and PCI with DES	PCI in ≥2 vessels	PCI in 1 vessel	Prior CABG. isolated LM. chronic occlusions. cardiogenic shock and staged PCI	532	366	NA
Shishehbor et al [27]	TARGET	2006	North America. Australia. Europe	RCT analysis	Propensity score matching	NSTE-ACS and PCI	PCI in ≥2 vessels	PCI in 1 vessel	Primary PCI. cardiogenic shock. creatinine >2.5 mg/dl. thrombocytopenia. bleeding diathesis. life-limiting conditions. staged PCI	4809	1302	NA
Shishehbor et al [5]		2007	United States	Observational registry	Multivariate analyses (Cox)	NSTE-ACS. multivessel disease and PCI with BMS		PCI in 1 vessel	Chronic occlusions. staged PCI. prior CABG	1240	1240	NA
Brener et al [9]	ACC-NCDR	2008	United States	Observational registry	Multivariate analyses (logistic regression)	NSTE-ACS. multivessel disease and PCI	PCI in ≥2 vessels	PCI in 1 vessel	Non-ACS patients. prior CABG. single vessel disease. staged PCI. and missing angiographic information	662463	105866	33
Kim et al [28]	KAMIR	2010	Korea	Observational registry	Multivariate analyses (Cox)	NSTEMI and multivessel disease			AMI	1919	1919	370
Mariani et al [15]	ROSAI	2001	Italy	Observational registry	Multivariate analyses (logistic regression)	Unstable angina and multivessel disease	PCI in all significant lesions	At least 1 residual stenosis >50%	Ongoing MI. previous PTCA or CABG	987	208	17
Palmer et al [31]		2004	United Kingdom	Observational registry	None	NSTE-ACS and multivessel disease	PCI in ≥2 vessels	PCI in 1 vessel	Prior CABG. LM lesion	219	151	13
Zapata et al [16]		2009	Argentine	Observational registry	Multivariate analyses (logistic regression)	NSTE-ACS. multivessel disease and PCI	PCI in ≥2 vessels	PCI in 1 vessel	STEMI. total chronic occlusions. staged PCI and prior CABG	1100	609	NA
Brener et al [25]	TACTICS-TIMI 18	2002	United States. Canada. South America. Europe	RCT analysis	None	NSTE-ACS and PCI	PCI in ≥2 vessels	PCI in 1 vessel	Non-culprit lesion only PCI	2220	427	NA
Hassanin et al [26]	Acuity	2014	Europe. United States and Canada	RCT analysis	Multivariate analyses (Cox)	Moderate or high risk NSTE-ACS and Multivessel disease	PCI in ≥2 vessels	PCI in 1 vessel	Staged PCI	13819	2864	NA

Abbreviations: ACS: acute coronary syndromes; PCI: percutaneous coronary interventions; CABG: coronary artery bypass grafting; LM: left main; NA: not available; NSTE-ACS: non-ST elevation acute coronary syndromes; DES: drug eluting stents; RCT: randomized clinical trial; BMS: bare metal stents; NSTEMI: non-ST elevation myocardial infarction; STEMI: ST elevation myocardial infarction.

Three studies (n = 4,456) were analyses post-hoc of randomized controlled trials [25–27], the remaining were retrospective analyses from observational registries (n = 113,229) [5,9,14–16,28–31]; there were no case control studies. Median count of stars from NOS was 6 (range 5 to 8). The observational registries involved one or more institutions from one country, whereas randomized controlled trials were international.

Two studies (n = 107,786) reported only in-hospital outcomes [9,14], for the remaining the median follow-up was 12 (range 6 to 36) months. Patients that met inclusion criteria and were analyzed represented from 4% to 75% of patients included in the original registries (Table 2). Only five studies reported the number of loss during follow-up [9,15,28,30,31].

**Table 2:** Characteristics of patients and follow-up.

Authors	Mean age, years		Male gender.,%		Diabetes, %		Previous MI, %		Chronic Kidney disease,%		Three vessel disease. %		Total occlusions. %		DES, %		B2-C type lesión, %		LVEF, %		Follow-up, months
	MV-PCI	CV-PCI	MV-PCI	CV-PCI	MV-PCI	CV-PCI	MV-PCI	CV-PCI	MV-PCI	CV-PCI	MV-PCI	CV-PCI	MV-PCI	CV-PCI	MV-PCI	CV-PCI	MV-PCI	CV-PCI	MV-PCI	CV-PCI	
Bauer et al [14]	65.0	67.0	69.3	73.4	28.6	30.0	33.8	34.1	5.3	6.8	29.8	26.2	12.3	16.1	45.6	34.2	NA	NA	NA	NA	In-hospital
Onuma et al [30]	64.6	64.1	30.9	30.3	20.1	18.5	45.2	52.0	NA	NA	NA	NA	NA	NA	56.3	59.9	84.3	72.3	NA	NA	36
Lee et al [29]	64.5	65.3	71.5	62.6	33.5	40.6	8.9	8.0	5.6	5.9	41.3	43.3	0.0	0.0	100	100	NA	NA	57.3	56.6	36
Shishehbor et al [27]	64.0	62.0	75.0	73.0	23.0	23.0	40.0	40.0	NA	NA	NA	NA	NA	NA	0.0	0.0	NA	NA	NA	NA	12
Shishehbor et al [5]	66.0	65.0	64.0	65.0	32.0	31.0	46.0	47.0	6.0	6.0	26.0	25.0	0.0	0.0	0.0	0.0	32.0	32.0	51.0	51.0	27
Brener et al [9]	65.0	66.0	64.4	64.7	31.5	31.9	25.2	29.3	5.1	5.9	NA	NA	13.8	24.2	NA	NA	22.8	25.6	55.0	55.0	In-hospital
Kim et al [28]	65.2	65.5	65.4	69.2	33.9	35.0	21.3	21.1	NA	NA	46.1	40.9	23.8	30.4	92.8	91.9	81.8	81.5	52.8	52.5	12
Mariani et al [15]	63.7	63.9	73.5	83.0	26.0	14.5	37.0	47.0	NA	NA	45.0	51.0	14.0	41.0	0.0	0.0	54.0	55.0	NA	NA	12
Palmer et al [31]	62.0	63.0	69.0	66.7	21.1	21.1	42.3	36.8	NA	NA	11.3	21.1	0.0	0.0	NA	NA	56.7	59.5	NA	NA	10
Zapata et al [16]	60.8	62.3	82.3	83.2	20.1	22.2	25.5	26.9	3.4	3.7	NA	NA	0.0	0.0	18.7	19.1	NA	NA	NA	NA	12
Brener et al [25]	62.0	62.0	71.0	67.0	30.0	27.0	44.0	43.0	NA	NA	59.0	54.0	13.0	15.0	NA	NA	NA	NA	55.0	54.0	6
Hassanin et al [26]	62.0	62.0	70.6	72.3	35.1	32.4	34.9	38.0	16	18	64.9	56	17.1	15.2	90.9	82.2	32.8	40.0	64.0	65.0	12

Abbreviations: DES: drug eluting stents; MI: myocardial infarction; LVEF: left ventricular ejection fraction; MV-PCI: multivessel percutaneous coronary intervention; CV-PCI: culprit-vessel percutaneous coronary intervention.

Table 2 also shows patients characteristics. Median age was 64.1 years, most patients were male, the median prevalence of smokers was 30.8%, diabetes mellitus was present in 29.3% of patients, and previous history of MI in 36.9%. Eight studies excluded patients with prior CABG [5,9,14–16,29–31].

There was a small excess of three-vessel disease among MV PCI, and lower prevalence of total chronic occlusions and complex lesions (B2 or C as defined by AHA/ACC classification). Mean left ventricular ejection fraction was normal and similar between groups across six studies that reported it [5,9,25,26,28,29], and it was also preserved in most patients when the threshold value was described in the study [14–16,27,31].

### Outcomes

**Fig 2:**
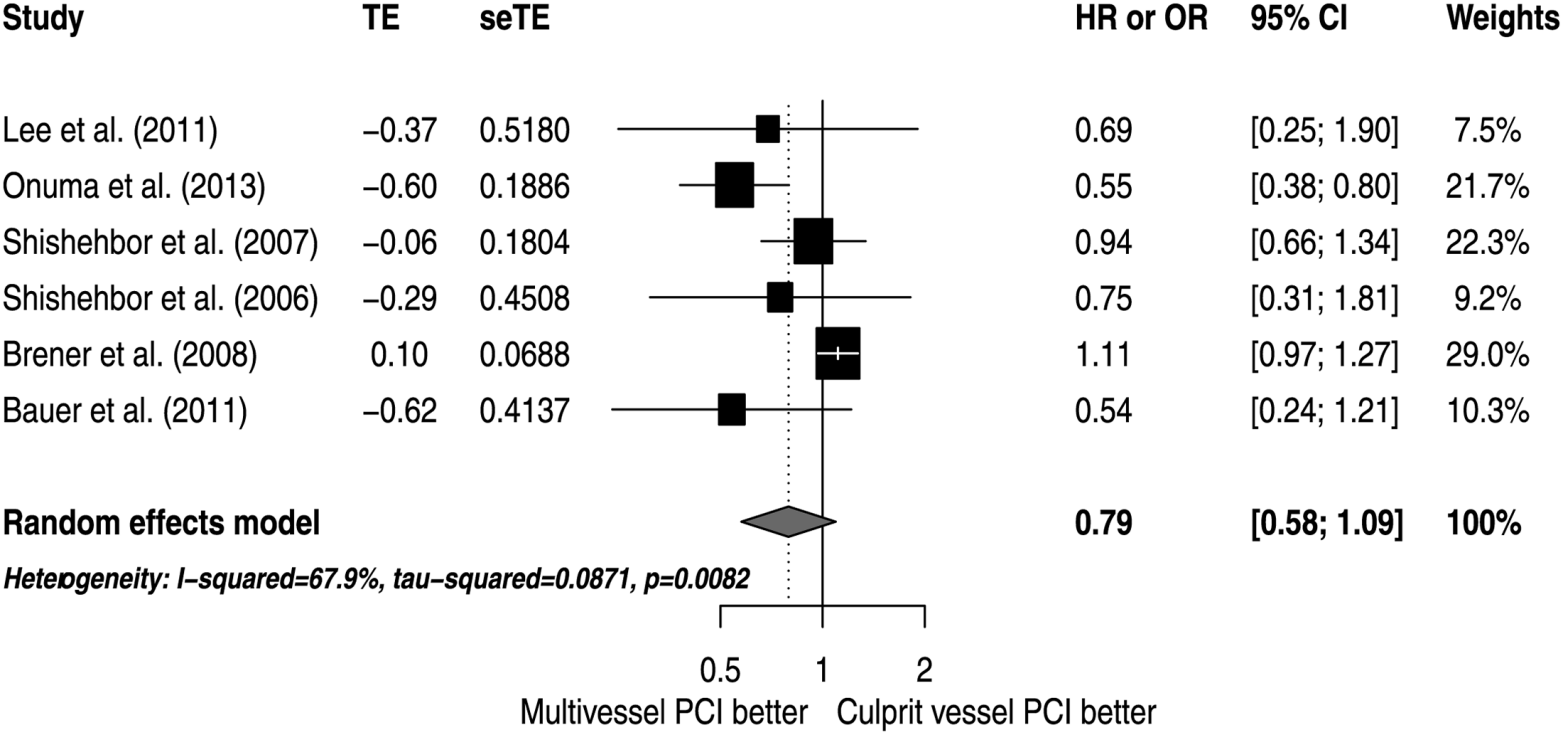
Effects of MV PCI versus CV PCI on mortality.

**Fig 3:**
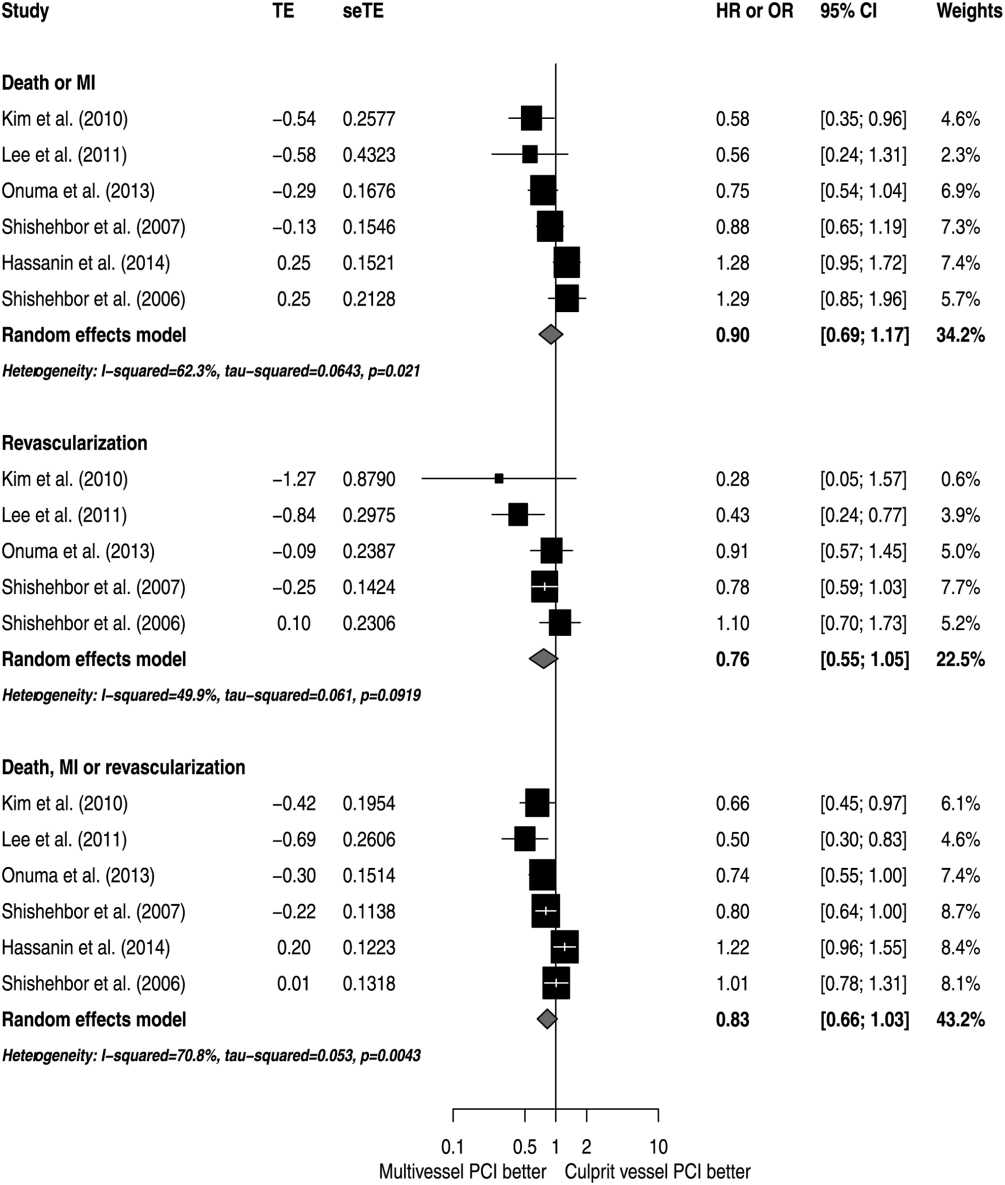
Effects of MV PCI versus CV PCI on secondary outcomes.

The analyses of adjusted estimators suggest that there were no significant differences in mortality risk (HR 0.79; 95% CI 0.58 to 1.09; I^2^ 67.9%), with moderate inconsistency (Fig 2). Also, there were no significant differences in the risk of death or MI (HR 0.90; 95% CI 0.69 to 1.17; I^2^ 62.3%), revascularization (HR 0.76; 95% CI 0.55 to 1.05; I^2^ 49.9%) or the combined outcome of death, MI or revascularization (HR 0.83; 95% CI 0.66 to 1.03; I^2^ 70.8%). All analyses exhibited a moderate degree of inconsistency (Fig 3).

**Fig 4:**
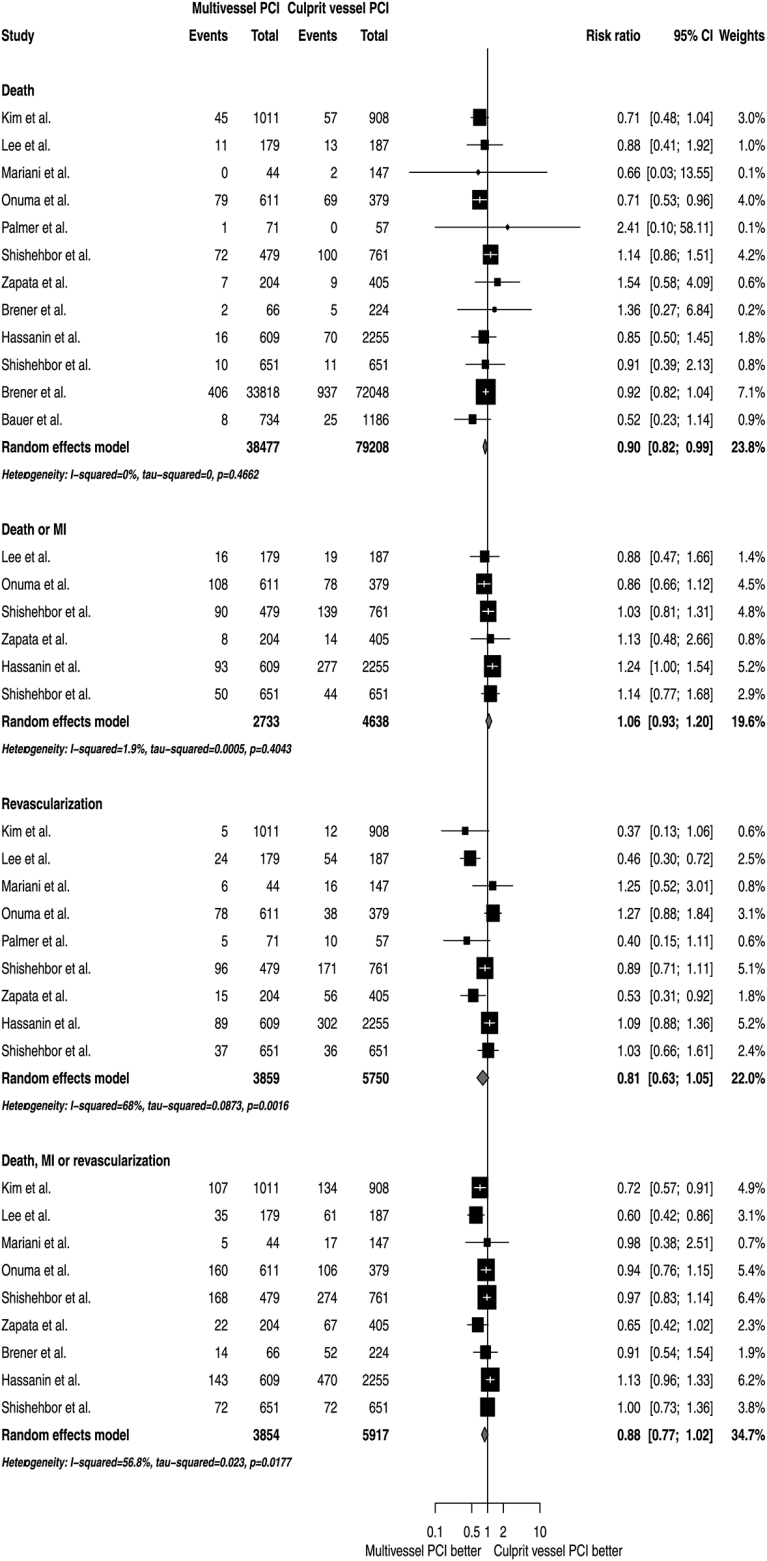
Unadjusted analyses of MV PCI versus CV PCI.

In unadjusted analyses, MV PCI was associated with a statistically significant reduction in the risk of death (RR 0.90; 95% CI 0.82 to 0.99), without heterogeneity across studies (I^2^ 0.0%). There were no statistically significant differences between revascularization strategies in the incidence of death or MI (RR 1.06; 95% CI 0.93 to 1.20; I^2^ 1.9%), revascularization (RR 0.81; 95% CI 0.63 to 1.05; I^2^ 68.0%) or the combined outcome of death, MI or revascularization (RR 0.88; 95% CI 0.77 to 1.02; I^2^ 56.8%) (Fig 4).

### Subgroup analyses

**Fig 5:**
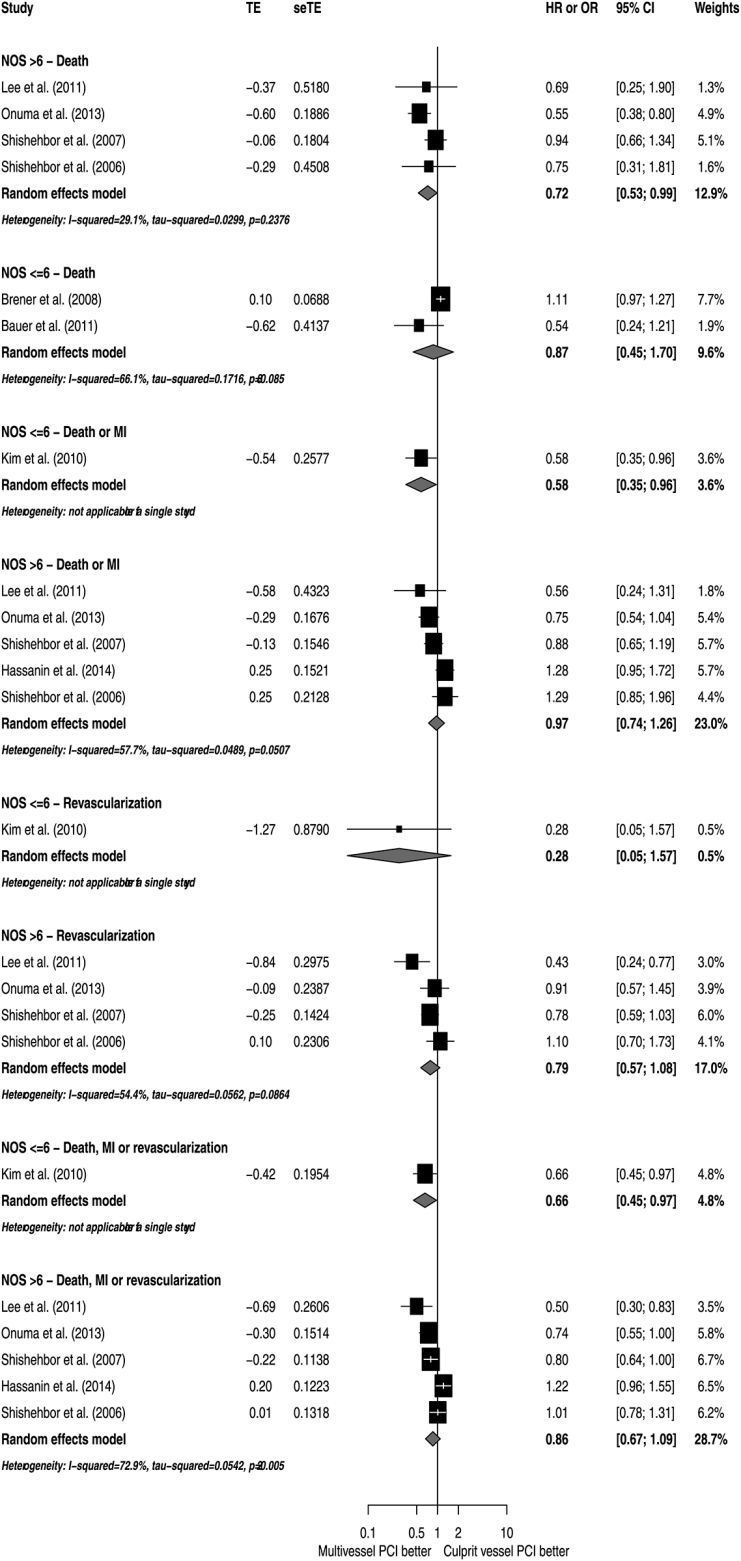
Subgroup analyses by quality of study report assessed by Newcastle-Ottawa Scale.

**Fig 6:**
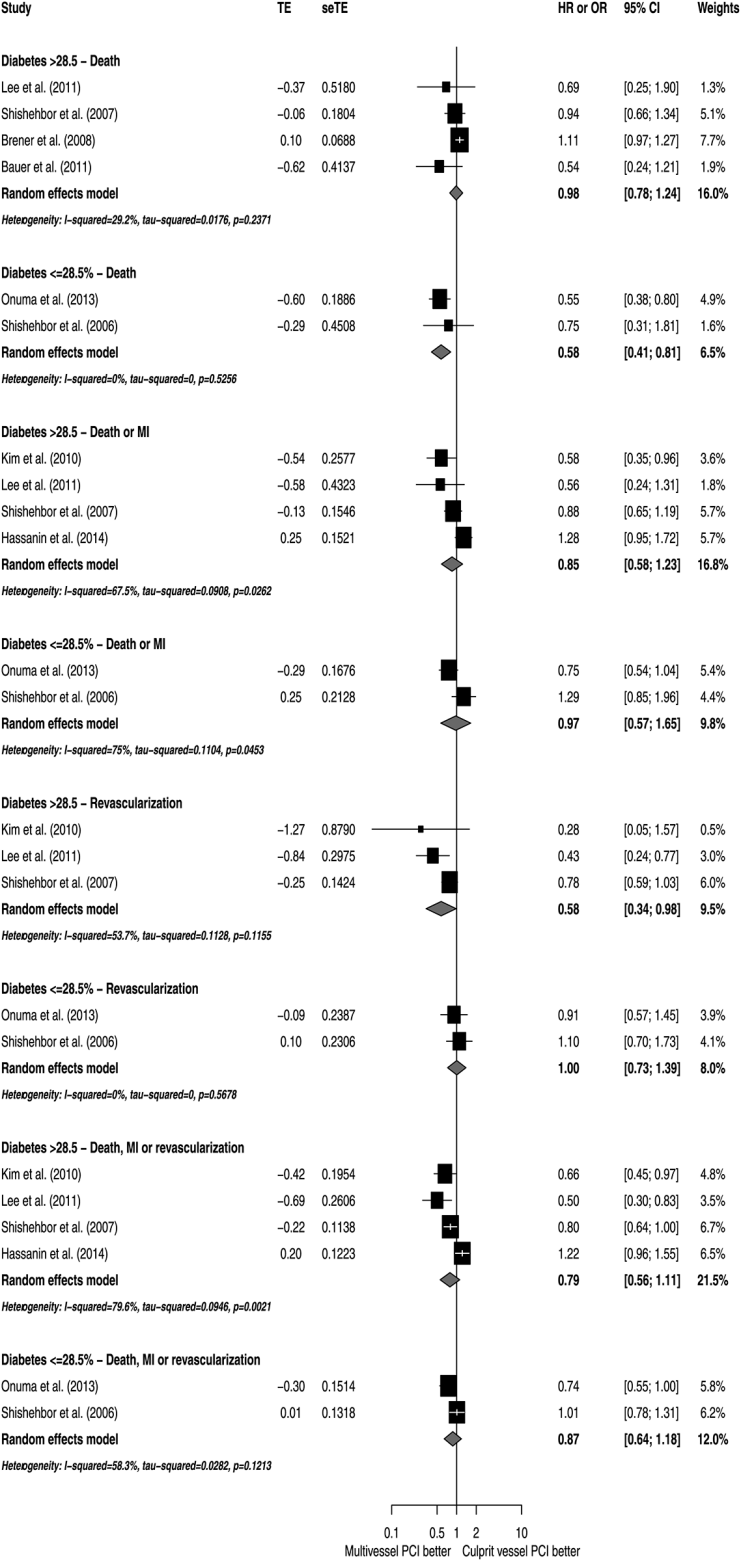
Subgroup analyses by follow-up.

**Fig 7:**
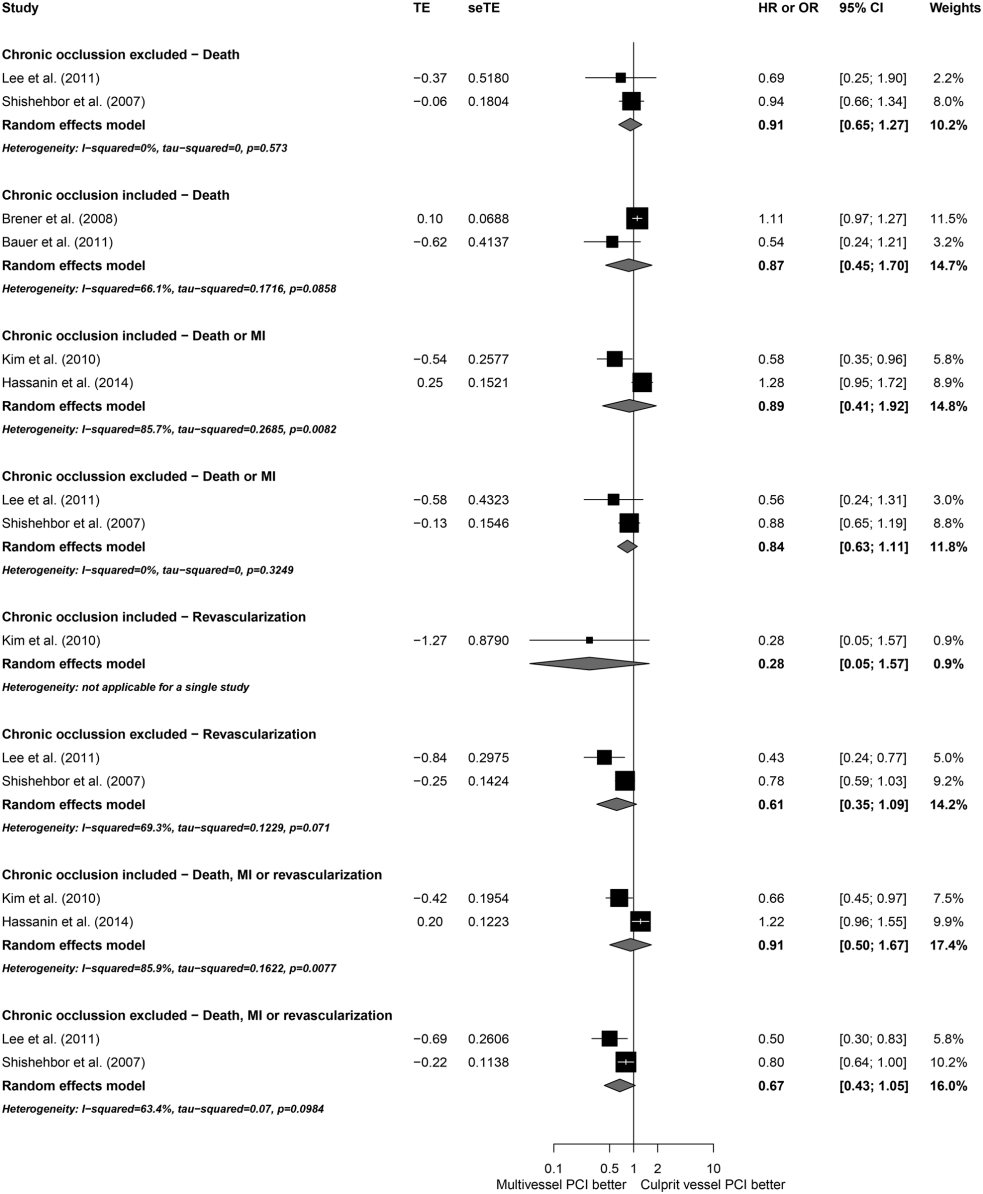
Subgroup analyses by DES use.

**Fig 8:**
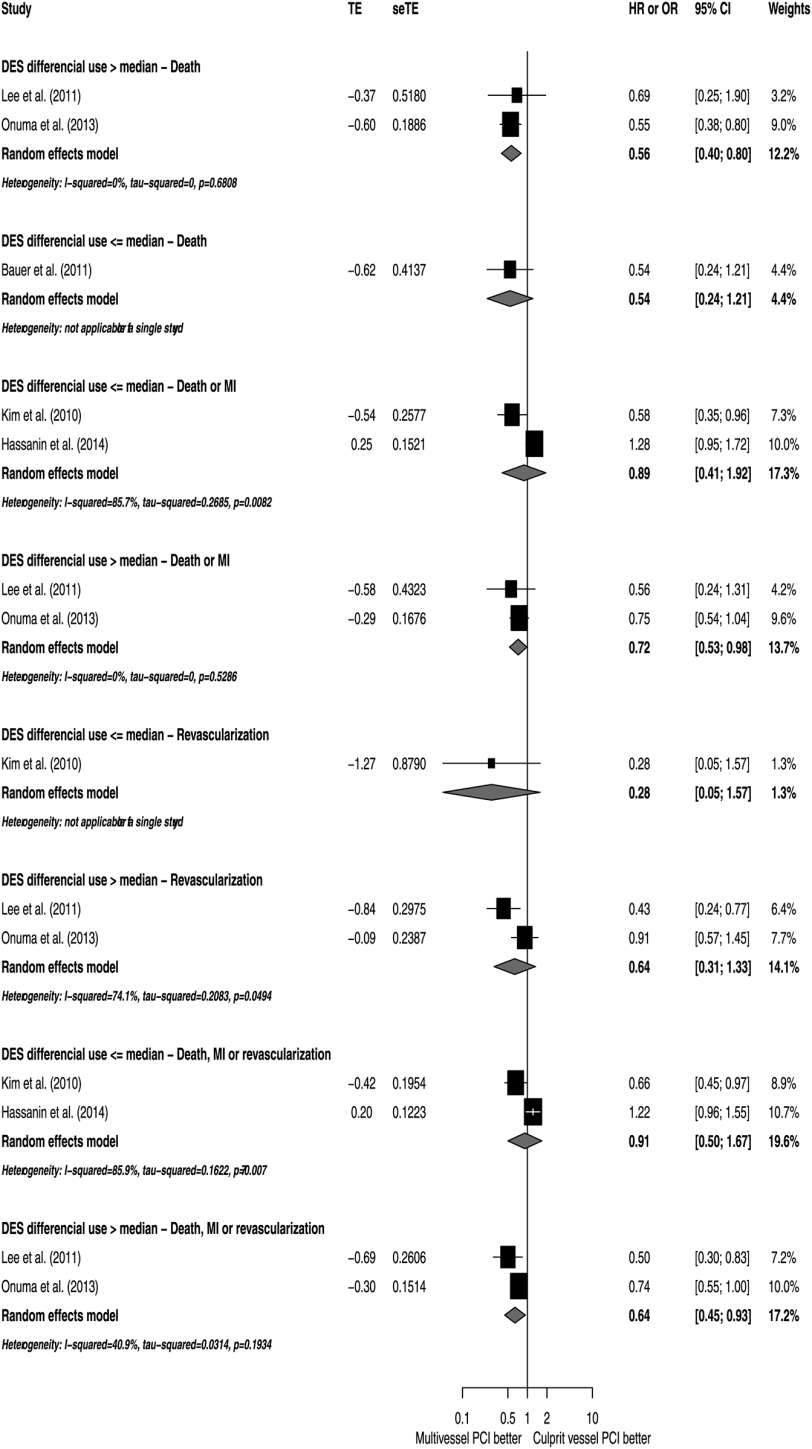
Subgroup analyses by diabetes prevalence.

**Fig 9:**
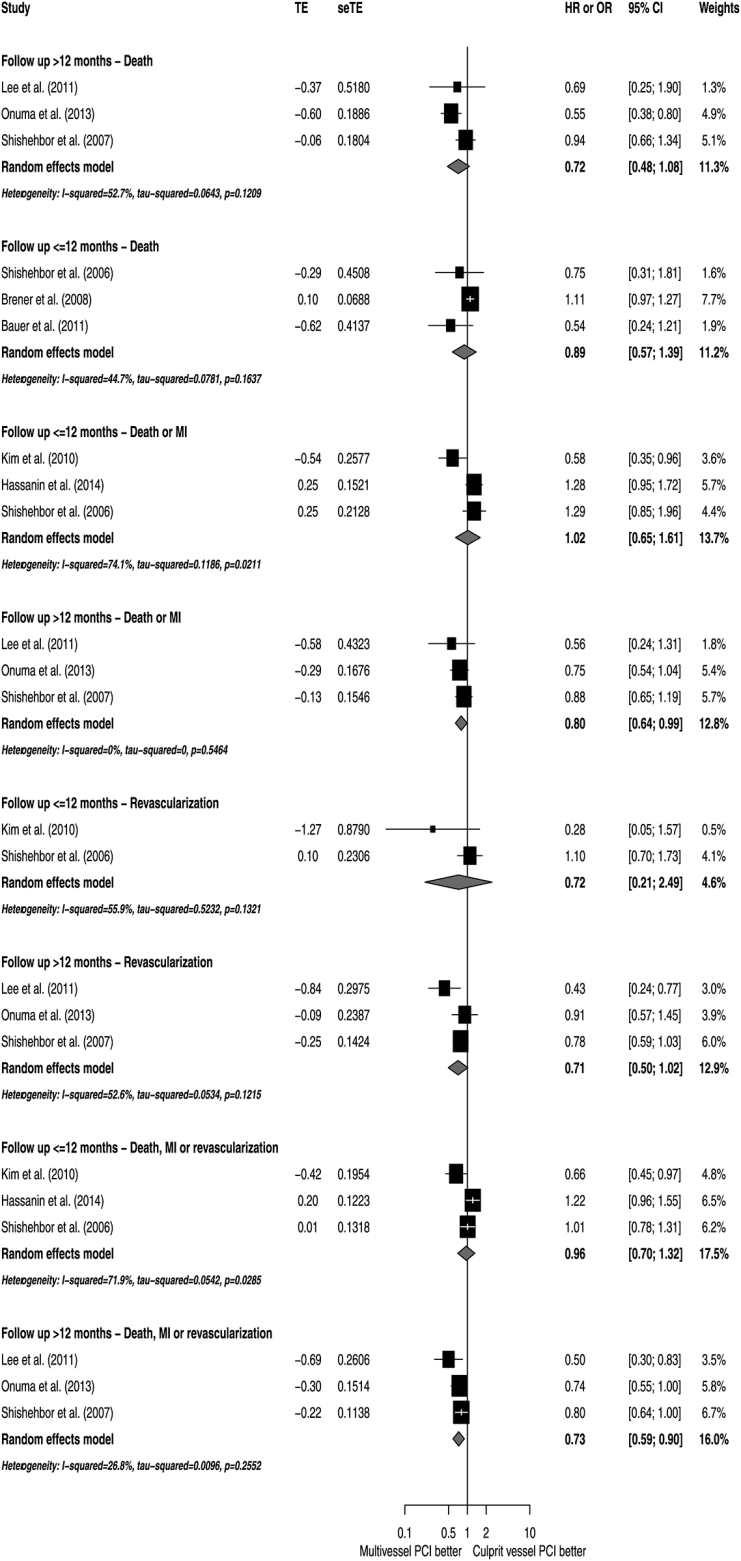
Subgroup analyses by chronic occlusions prevalence.

**Fig 10:**
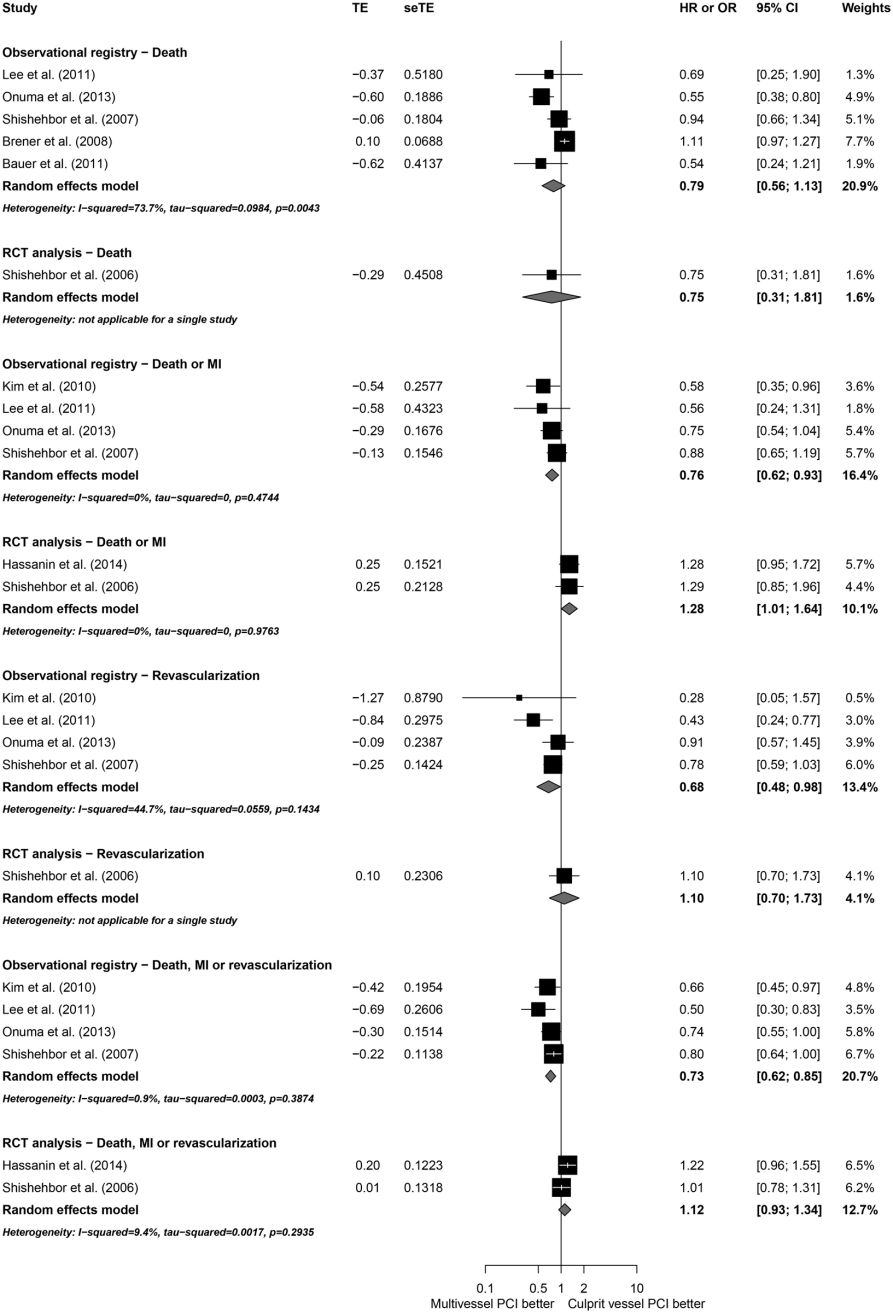
Subgroup analyses by studies design.

To explore potential sources of heterogeneity, subgroup analyses according to NOS, study designs, patient characteristics and utilization of DES were conducted (Figs 5–9). Analyzing separately the studies by their design (RCT *post-hoc* analysis versus Observational registries) the inconsistency of the analyses were reduced in terms of death or MI, revascularization and death, MI or revascularization (Fig 10); however, there was no variable that explained inconsistency across estimators of effect on mortality.

### Sensitivity analysis

**Fig 11:**
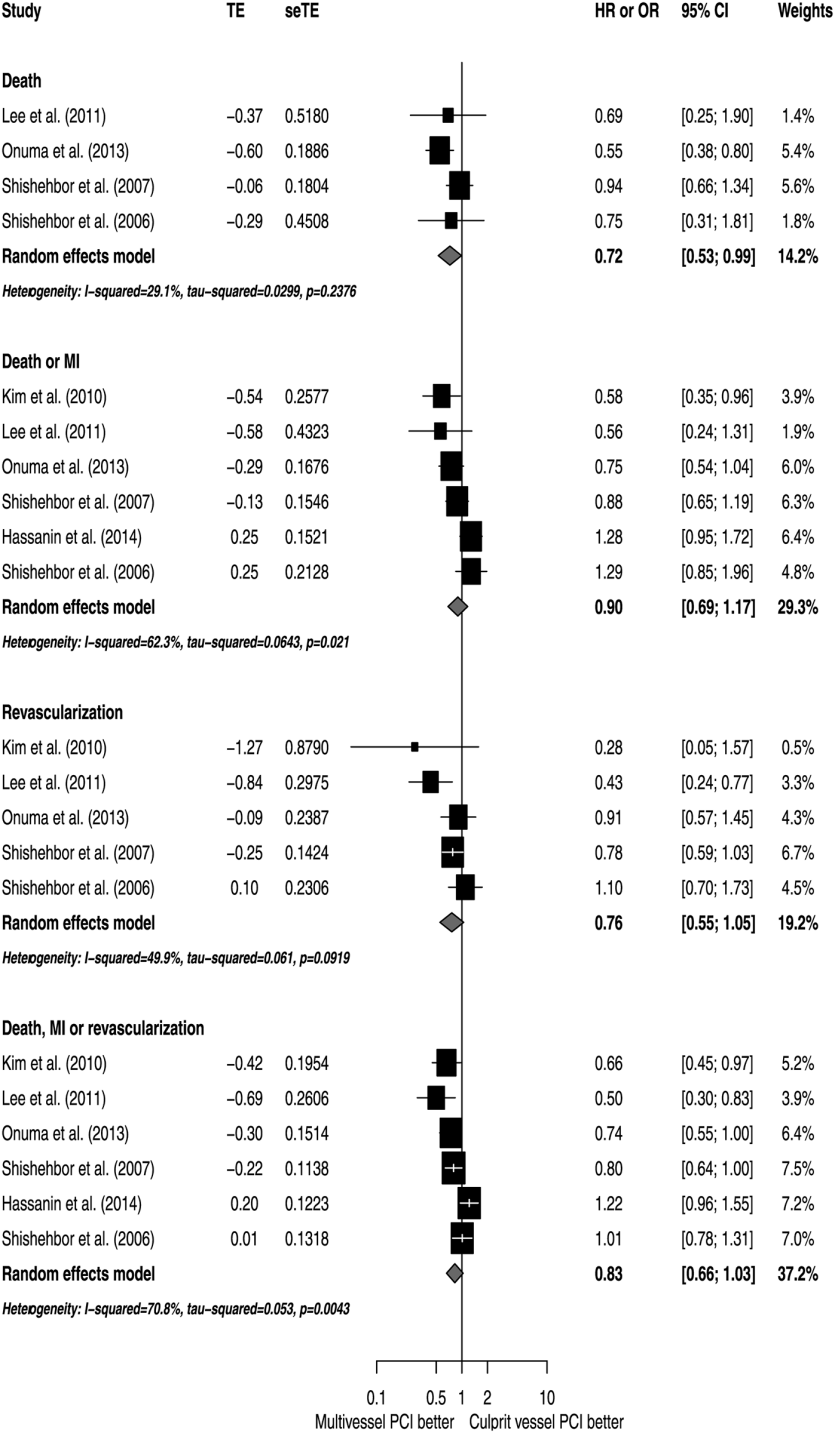
Sensitivity analyses with odds ratios transformation to risk ratios.

**Fig 12:**
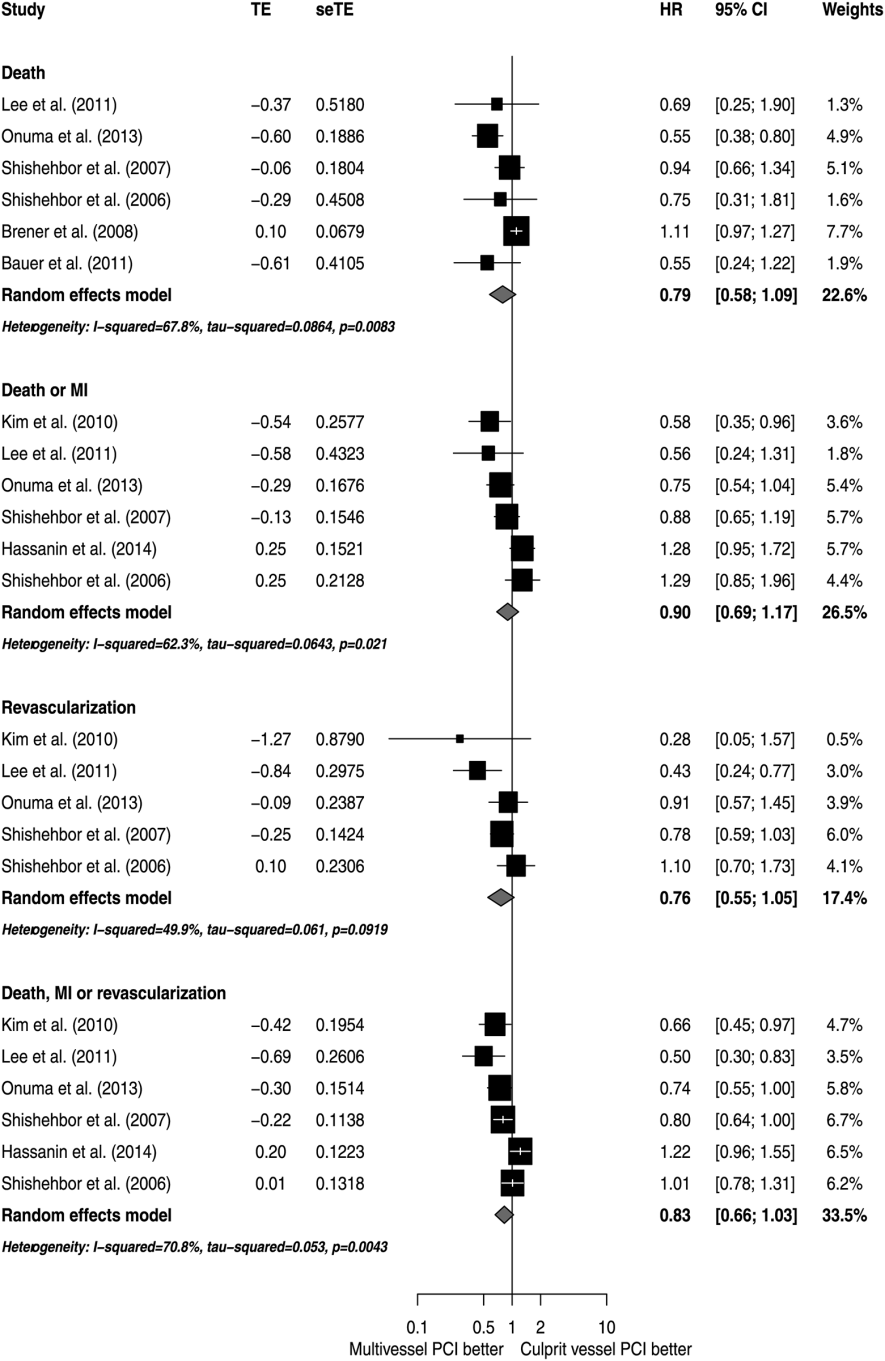
Sensitivity analyses excluding studies with follow-up limited to initial hospitalization.

Results were almost identical after correction of effect estimators presented as OR (Fig 11). After exclusion of studies that reported only in-hospital outcomes, mortality analysis suggested a benefit from MV PCI with a lower level of inconsistency (Fig 12).

### Publication bias

**Fig 13:**
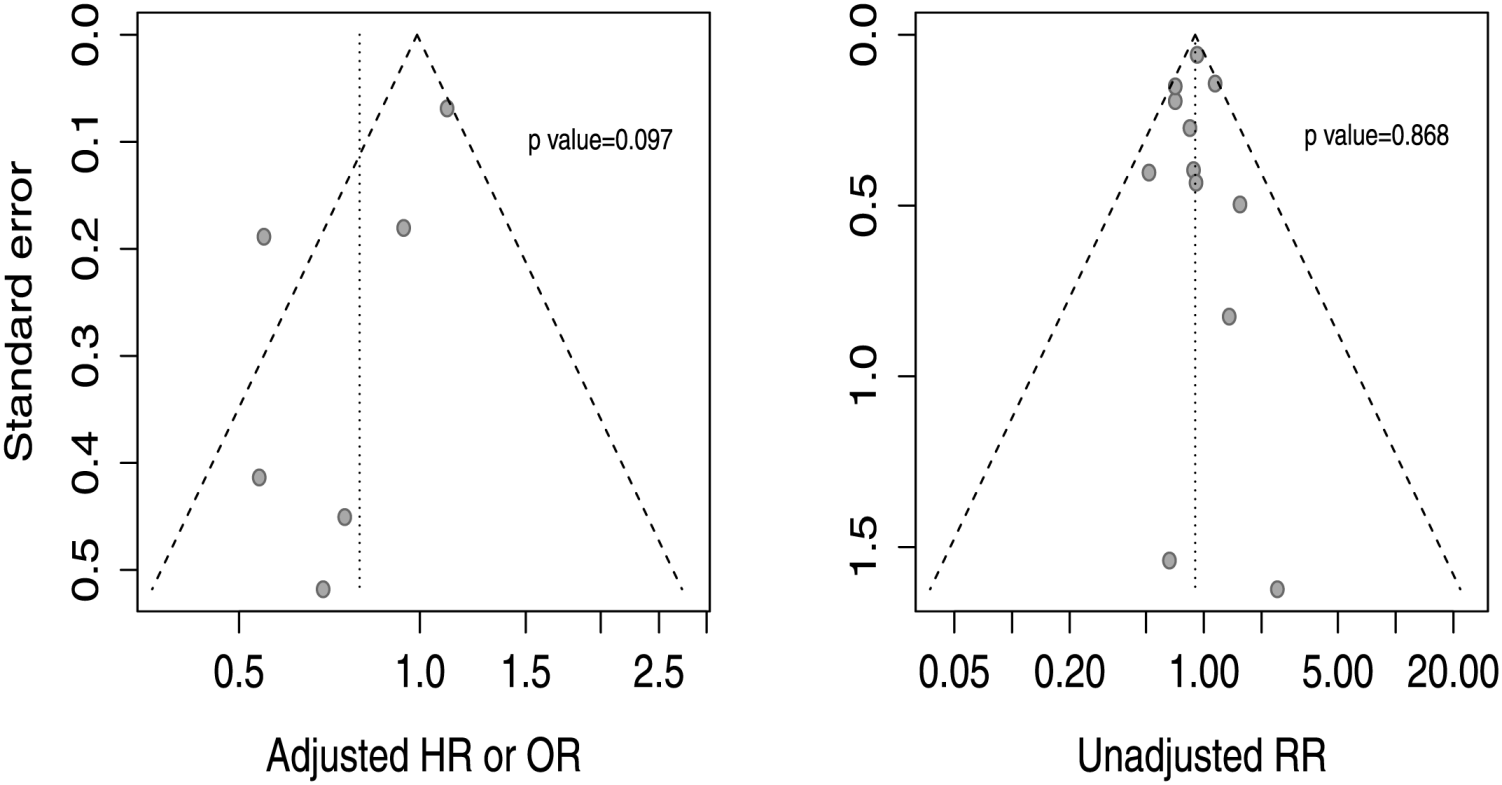
Funnel plots of adjusted (13A) and unadjusted (13B) estimates.

Visual inspection of funnel plot suggested asymmetry among studies that had reported adjusted estimates of effect (Fig 13A), and the formal evaluation indicated the presence of publication bias (p = 0.097). Fig 13B shows funnel plot constructed with unadjusted effect estimates with no evidence of publication bias (p = 0.868); differences between both plots suggested differential reporting of adjusted analyses.

## Discussion

The results of present meta-analysis, based on observational studies that compared MV PCI versus CV PCI among NSTE-ACS patients with multivessel disease, suggested that there were no significant differences between both revascularization strategies.

Current clinical practice guidelines for the management of NSTE-ACS indicate that MV PCI could be reasonable in patients undergoing coronary revascularization as part of the treatment strategy [2]. This recommendation is based on reports of studies suggesting that MV PCI is a safe intervention and that it reduces the need for revascularization procedures during follow up [6, 9, 16, 25, 31]. However, this meta-analysis does not confirm the reduction of future revascularization procedures during follow up. Furthermore, regarding safety data of MV PCI, is important to notice that, although in overall results showed no significant differences between both strategies in mortality, MI or revascularization risks, these results are heterogeneous and part of the heterogeneity is controlled with stratified analyses by study design, such that most rigorous data (those from post-hoc analyses of RCT) suggest an increase of death, MI or MACE risks with MV PCI. Hence, according these results, CV PCI should be the revascularization strategy preferred for most NSTE-ACS patients with multivessel disease undergoing PCI, excepting possibly those without a clearly identifiable culprit-vessel in whom a more extensive revascularization could be a better strategy.

Systematic monitoring for periprocedural MI might explain the results heterogeneity between observational registries and post-hoc analyses of RCT. Higher risk of MI after MV PCI has been related to distal embolization, side branch closure and stent thrombosis, which could be heightened after multiple stent deployment in a pro-inflammatory and pro-thrombotic environment [6,32].

The analyses have several limitations that should be considered at interpreting the results. First, this is a meta-analysis of observational studies and, although adjusted estimators of effect were used to minimize biases, some degree of residual confounding is possible [10]. In all studies, treatment groups were defined after PCI, such that patients in whom originally planned strategy was MV PCI, but received only one-vessel PCI because technical or anatomic factors were classified as CV PCI, which could bias results against CV PCI. Furthermore, most studies derived from analyses from larger datasets, leaving the possibility of selection bias. Finally, there is evidence of publication bias, with smaller studies suggesting more benefits for MV PCI.

In conclusion, the results of this meta-analysis suggests that routine MV PCI in NSTE-ACS patients with multivessel disease is not superior to CV PCI and, that there is evidence that it could be not equally as safe. Since, there is a high prevalence of multivessel disease among NSTE-ACS patients and the available evidence has multiple limitations, randomized controlled trials evaluating safety and effectiveness of MV PCI in this setting are needed.

## Supporting Information

S1 Data Database for the mortality (main) analyses. (CSV)

S1 MOOSE Checklist MOOSE Checklist. (DOCX)
