# Supplementary material for: Multivessel versus Single Vessel Angioplasty in Non-ST Elevation Acute Coronary Syndromes: A Systematic Review and Metaanalysis
Source: PLoS One. 2016 Feb 17;11(2):e0148756. doi: 10.1371/journal.pone.0148756 (PMC4757575; doi:10.1371/journal.pone.0148756)
Supplement: S1 MOOSE Checklist — (DOCX) [file pone.0148756.s002.docx]

**MOOSE Checklist**

From: [Donna F. Stroup](http://jama.ama-assn.org/search?author1=Donna+F.+Stroup&sortspec=date&submit=Submit), PhD, MSc; [Jesse A. Berlin](http://jama.ama-assn.org/search?author1=Jesse+A.+Berlin&sortspec=date&submit=Submit), ScD; [Sally C. Morton](http://jama.ama-assn.org/search?author1=Sally+C.+Morton&sortspec=date&submit=Submit), PhD; [Ingram Olkin](http://jama.ama-assn.org/search?author1=Ingram+Olkin&sortspec=date&submit=Submit), PhD; [G. David Williamson](http://jama.ama-assn.org/search?author1=G.+David+Williamson&sortspec=date&submit=Submit), PhD; [Drummond Rennie](http://jama.ama-assn.org/search?author1=Drummond+Rennie&sortspec=date&submit=Submit), MD; [David Moher](http://jama.ama-assn.org/search?author1=David+Moher&sortspec=date&submit=Submit), MSc; [Betsy J. Becker](http://jama.ama-assn.org/search?author1=Betsy+J.+Becker&sortspec=date&submit=Submit), PhD; [Theresa Ann Sipe](http://jama.ama-assn.org/search?author1=Theresa+Ann+Sipe&sortspec=date&submit=Submit), PhD; [Stephen B. Thacker](http://jama.ama-assn.org/search?author1=Stephen+B.+Thacker&sortspec=date&submit=Submit), MD, MSc; for the Meta-analysis Of Observational Studies in Epidemiology (MOOSE) Group. **Meta-analysis of Observational Studies in Epidemiology. A Proposal for Reporting** JAMA. 2000;283(15):2008-2012. doi: 10.1001/jama.283.15.2008

|  | Reported on page | Comments |
| --- | --- | --- |
| **Reporting of background should include** | | |
| Problem definition | 5 | Lines 2 to 13. |
| Hypothesis statement | 5 | Lines 20 to 22. |
| Description of study outcomes | 7 and 8 | Lines 22,23 (page 7) and 1 and 2 (page 8). |
| Type of exposure or intervention used | 24 | Table 1. |
| Type of study designs used | 7 | Lines 7 and 8. |
| Study population | 6 | Lines 6 to 9; and line 13. |
| **Reporting of search strategy should include** | | |
| Qualifications of searchers (eg librarians and investigators) | 6 | Line 17, “we” is used since search was conducted by medical doctors. |
| Search strategy, including time period used in the synthesis and key words | 6 | Lines 17 to 24. |
| Effort to include all available studies, including contact with authors | 6 | Lines 10 to 12 (there were no restrictions), we tried to contact one of de authors for clarification of effect estimate (Dr. Zapata) and got no response. |
| Databases and registries searched | 6 | Lines 17 and 18. |
| Search software used, name and version, including special features used (eg explosion) | NA | We searched electronic databases. |
| Use of hand searching (eg reference lists of obtained articles) | 6 | Lines 23 and 24. |
| List of citations located and those excluded, including justification | 23 | Figure 1. |
| Method of addressing articles published in languages other than English | NA | There were no other languages articles located. |
| Method of handling abstracts and unpublished studies | NA | We did not found unpublished studies. |
| Description of any contact with authors | 7 | Lines 1, 2. |
| **Reporting of methods should include** | | |
| Description of relevance or appropriateness of studies assembled for assessing the hypothesis to be tested | 10 |  |
| Rationale for the selection and coding of data (eg sound clinical principles or convenience) | 7 and 8 |  |
| Documentation of how data were classified and coded (eg multiple raters, blinding and interrater reliability) | 7 |  |
| Assessment of confounding (eg comparability of cases and controls in studies where appropriate) | 8 |  |
| Assessment of study quality, including blinding of quality assessors, stratification or regression on possible predictors of study results | 7 |  |
| Assessment of heterogeneity | 8 |  |
| Description of statistical methods (eg complete description of fixed or random effects models, justification of whether the chosen models account for predictors of study results, dose-response models, or cumulative meta-analysis) in sufficient detail to be replicated | 8 |  |
| Provision of appropriate tables and graphics | 24-39 |  |
| **Reporting of results should include** | | |
| Graphic summarizing individual study estimates and overall estimate | 27-28 | Figure 2, figure 3 |
| Table giving descriptive information for each study included | 24 and 25 | Table 1 and table 2 |
| Results of sensitivity testing (eg subgroup analysis) | 11 and 12 | Figures 4, supplemental figures. |
| Indication of statistical uncertainty of findings | 11 | Figures 2 and 3. |
| **Reporting of discussion should include** | | |
| Quantitative assessment of bias (eg publication bias) | 12 | Figure 5. |
| Justification for exclusion (eg exclusion of non-English language citations) | 6 |  |
| Assessment of quality of included studies | 7, 13 and 14. |  |
| **Reporting of conclusions should include** | | |
| Consideration of alternative explanations for observed results | 14 |  |
| Generalization of the conclusions (eg appropriate for the data presented and within the domain of the literature review) | 14 |  |
| Guidelines for future research | 14 |  |
| Disclosure of funding source | 15 |  |

Transcribed from the original paper within the Support Unit for Research Evidence (SURE), Cardiff University, United Kingdom. February 2011.
